# Analytical Prediction of the Spatiotemporal Distribution of Chemoattractants around Their Source: Theory and Application to Complement-Mediated Chemotaxis

**DOI:** 10.3389/fimmu.2017.00578

**Published:** 2017-05-26

**Authors:** Volkmar Heinrich, Wooten D. Simpson, Emmet A. Francis

**Affiliations:** ^1^Department of Biomedical Engineering, University of California at Davis, Davis, CA, United States

**Keywords:** chemotaxis, human neutrophil, complement, anaphylatoxin, reaction–diffusion, mathematical model, single-cell, host–pathogen

## Abstract

The ability of motile immune cells to detect and follow gradients of chemoattractant is critical to numerous vital functions, including their recruitment to sites of infection and—in emerging immunotherapeutic applications—to malignant tumors. Facilitated by a multitude of chemotactic receptors, the cells navigate a maze of stimuli to home in on their target. Distinct chemotactic processes direct this navigation at particular times and cell-target distances. The expedient coordination of this spatiotemporal hierarchy of chemotactic stages is the central element of a key paradigm of immunotaxis. Understanding this hierarchy is an enormous interdisciplinary challenge that requires, among others, quantitative insight into the shape, range, and dynamics of the profiles of chemoattractants around their sources. We here present a closed-form solution to a diffusion–reaction problem that describes the evolution of the concentration gradient of chemoattractant under various conditions. Our ready-to-use mathematical prescription captures many biological situations reasonably well and can be explored with standard graphing software, making it a valuable resource for every researcher studying chemotaxis. We here apply this mathematical model to characterize the chemoattractant cloud of anaphylatoxins that forms around bacterial and fungal pathogens in the presence of host serum. We analyze the spatial reach, rate of formation, and rate of dispersal of this locator cloud under realistic physiological conditions. Our analysis predicts that simply being small is an effective protective strategy of pathogens against complement-mediated discovery by host immune cells over moderate-to-large distances. Leveraging our predictions against single-cell, pure-chemotaxis experiments that use human immune cells as biosensors, we are able to explain the limited distance over which the cells recognize microbes. We conclude that complement-mediated chemotaxis is a universal, but short-range, homing mechanism by which chemotaxing immune cells can implement a last-minute course correction toward pathogenic microbes. Thus, the integration of theory and experiments provides a sound mechanistic explanation of the primary role of complement-mediated chemotaxis within the hierarchy of immunotaxis, and why other chemotactic processes are required for the successful recruitment of immune cells over large distances.

## Introduction

### Paradigm of the Spatiotemporal Organization of Immunotaxis

It is well known that host-cell-produced chemoattractants play a key role in the recruitment of immune cells to sites of infection, trauma, or inflammation. (We will henceforth use the term “chemokine” to summarily denote chemoattractants released by host cells, which also encompasses non-peptides like chemoattractant leukotrienes.) But because these endogenous chemicals originate from host cells, they cannot guide the responding cells to invasive pathogenic targets. In fact, if their gradients provided the only directional cues for chemotaxing immune cells like neutrophils, the neutrophils would not be able to participate in pathogen-specific defenses requiring precontact recognition of invaders. Worse, they would ultimately target the host cells that generate such chemokine gradients.

Evidently, chemotaxing immune cells require additional directional guidance via chemicals that emanate from the surfaces of microbes rather than host cells. Consequently, there must exist a type of chemotaxis that is not guided by chemokines. This pathogen-directed chemotaxis must be able to subjugate concurrent chemotactic stimuli that do not originate from the actual targets of the responding immune cells.

Having accepted that there is a route of chemotaxis that employs exceptionally potent chemoattractants and guides immune cells directly to their target, a new question arises: why then are chemokines needed? A potentially useful role of chemokines is the mobilization of reinforcements to help fight off invaders. But how effective can this support be when chemokines fail to direct the newly recruited cells toward the invaders, and when desensitizing mechanisms are needed to deter the reinforcements from attacking the chemokine-producing cells of their own host?

**Figure 1:**
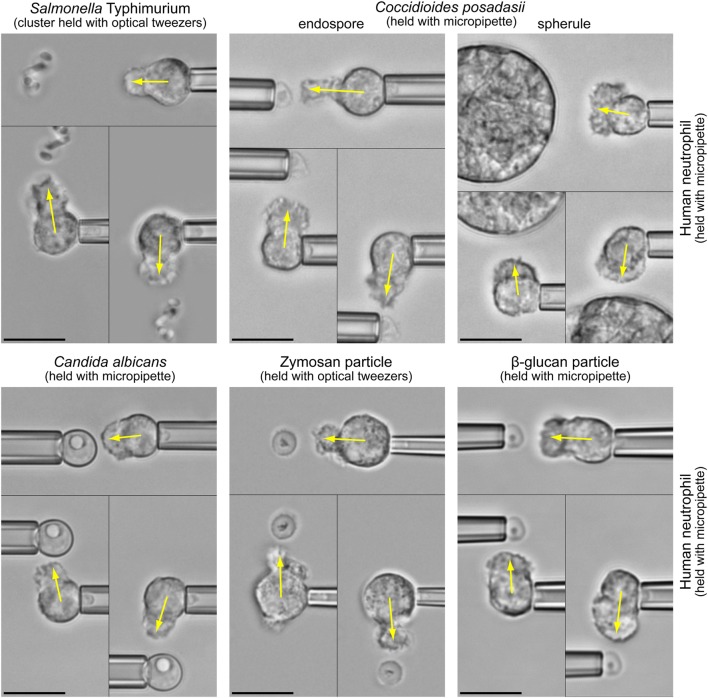
**Highly controlled one-on-one encounters between immune cells and microbes**. Micropipettes or optical tweezers are used to maneuver bacterial and fungal pathogens, as well as surrogate particles such as zymosan (made from cell walls of yeast) or β-glucan particles, into the proximity of initially quiescent, pipette-held human neutrophils without touching the cells. The non-adherent neutrophils react by forming pseudopods directed toward the nearby targets (indicated by arrows). We usually triple-check the specificity of the response by repositioning the target to different sides of the cell (see also Video S1 in Supplementary Material). In the shown experiments, the neutrophils respond to the cloud of anaphylatoxins (in particular C5a) that forms around the target particles in the presence of autologous serum. All scale bars denote 10 μm.

**Figure 2:**
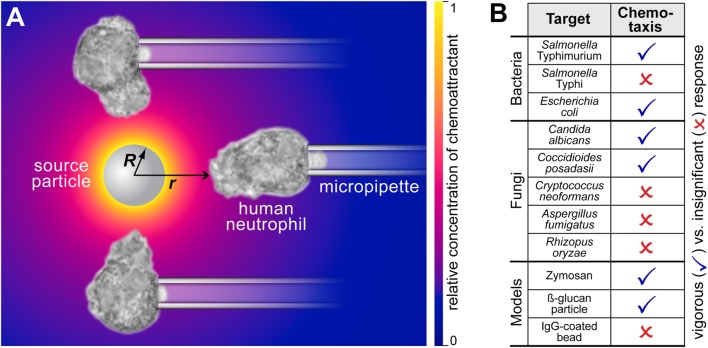
**Complement-mediated chemotactic recognition of pathogen surfaces by human neutrophils**. **(A)** The mechanism by which the neutrophils detect and locate pathogenic surfaces is complement-mediated chemotaxis. The host’s complement system assembles enzymes on the surface of foreign target particles. Enzymes like the C5 convertase cleave highly potent chemoattractant peptides such as C5a from serum proteins and release them. The resulting concentration of chemoattractant is shown as a density plot using a yellow-to-blue color gradient. Neutrophils detect these anaphylatoxins and respond by forming chemotactic pseudopods. The sketch also defines the geometric parameters *R* and *r*. **(B)** Overview of the aptitude of human neutrophils to recognize various bacterial, fungal, and model pathogens by complement-mediated chemotaxis. This cumulative table summarizes the results of previous studies (1–4). The list compares human-immune-cell interactions with 11 different targets under identical, near-physiological conditions. All experiments were performed with unprimed human neutrophils in the presence of 10–20% autologous donor serum. The single-cell, pure-chemotaxis experiments clearly discriminate between pathogenic targets that elicit a vigorous response from a distance, and those that are protected against complement-mediated chemotactic recognition. An example of a negative response (in the case of *C. neoformans*) is included in Video S1 in Supplementary Material.

The fact that both types of chemoattractant—host-cell-produced and pathogen-directed—are indeed important has recently been illuminated by the juxtaposition of *in vitro* studies of pathogen-directed chemotaxis and clinical manifestations of related infections (1, 2). The chemotaxis experiments (illustrated in Figure 1 and Video S1 in Supplementary Material) confronted individual human neutrophils with real-world pathogens and surrogate particles. The experimental design (Figure 2A) precluded the involvement of cell-substrate adhesion or chemokines in guiding the chemotaxing cells. Figure 2B compiles the results of such pure-chemotaxis tests performed with 11 different targets under otherwise identical conditions (1–4). Intriguingly, some of the *in vitro* results were reported to be inconsistent with expectations based on clinical observations. For example, compared to the well-known mobilization of neutrophils in candidiasis, neutrophils play a much smaller role in coccidioidomycosis (or Valley fever). However, the single-cell chemotaxis experiments did not reproduce this trend. Instead, the *in vitro* responses of neutrophils to both parasitic forms of *Coccidioides posadasii* (endospores and spherules; Figure 1) closely mirrored the vigorous responses to *Candida albicans* and zymosan (2). Thus, although *C. posadasii* elicits a strong chemotactic response by nearby neutrophils, the fungus appears to be able to subdue long-range recruitment of these cells *in vivo*.

Clearly, the *spatial range* of an interaction, along with the interaction strength, is critical to its physiological function and relevance. The pure-chemotaxis experiments (such as shown in Figure 1) revealed that in the absence of adhesion and chemokines, positive chemotactic responses occurred only over *small* cell-target distances. Accordingly, chemokines must be responsible, and are thus required, for *long-range* mobilization and recruitment of immune cells.

These considerations lead to the conclusion that intact immunotaxis—the successful recruitment of immune cells—comprises a well-orchestrated sequence of distinct chemotactic processes. Different sets of chemoattractant mediate different stages of this sequence. Each stage occupies a specific slot within the timeline of the cellular response and controls the cell motion over a particular range of cell-target distances. We note that this *spatiotemporal* hierarchy is different from the signaling hierarchy of chemoattractant-specific pathways addressed in Ref. (5, 6).

The physiological implementation of this *paradigm of immunotaxis* is incredibly complex, but its basic immunobiology is reasonably well understood (2, 5–15). In broad strokes, after neutrophils are captured from the blood stream by endothelial adhesion molecules, they extravasate into the surrounding tissue and follow chemokines secreted by macrophages or other early responders to an infection. Closer to the site of infection, intermediate chemokines like interleukin 8 or leukotriene B4 predominantly guide the directional motion of the cells. Finally, “end-target” or “terminal target-derived” chemoattractants like anaphylatoxins or N-formylated peptides redirect the chemotaxing cells toward the actual pathogens. [For recent reviews, see Ref. (14, 15).]

### Immunology beyond Immunobiology: A Sprawling Frontier

The analysis presented in this paper aims to extend our quantitative understanding of immunotaxis. Its conceptual home is the largely unexplored space of subdisciplines of immunology that complement immunobiology. [For perspectives discussing this sprawling interdisciplinary frontier, see Ref. (16, 17).] We here address the original cause of an immune cell’s decision to follow one chemoattractant over another, i.e., the local composition of chemical stimuli and, possibly, their gradients. In other words (and excluding time-dependent desensitization effects), changes in the chemotactic cell response are ultimately caused by changes in the *relative strengths* of different chemotactic stimuli encountered at the cell surface. Therefore, accurate knowledge of the local concentrations of chemoattractants is absolutely essential for predictive assertions about immunotaxis.

It seems difficult to extract this kind of information from the popular and highly instructive under-agarose assay (5, 6, 18). Although micromolar concentrations of folate have recently been quantified for a one-well assay (19), the same technique is unlikely to be applicable to the picomolar levels of chemoattractants used with immune cells (5, 6). To resolve these and other uncertainties, a more reductionist approach is needed, preferably one that also allows us to mimic serum interactions with chemoattractants, such as the deactivation of the anaphylatoxin C5a (20, 21) by carboxypeptidases (22). Complementing traditional bulk assays, experiments such as shown in Figure 1 are generally more amenable to quantitative predictions of the concentration profile of chemoattractants around their sources.

The main objective of the theoretical part of this paper is to translate a realistic scenario of chemotactic-gradient formation into an appropriate mathematical form, and to find an analytical solution of the resulting diffusion-reaction problem. Because our closed-form theoretical prescription can easily be implement using standard graphing software (even Microsoft Excel), it should be a valuable resource for anyone interested in chemotaxis research. Among others, it takes the guesswork out of questions like, what is the shape of a chemoattractant gradient? How non-linear is it? How steep is it at some distance from a source? What defines the gradient shape? How is it affected by physiological or experimental conditions? How does the concentration profile change over time? How quickly does it approach a steady state? How long does the chemoattractant linger after its source has been removed? How far from a source can we expect an immune cell to sense the source? How many chemoattractant molecules are available as potential GPCR ligands in the immediate proximity of the cell, and how does this number vary along the cell surface? How many GPCRs are, on average, ligated at the moment when a cellular response first commences?

The applied section of this study demonstrates how our theory can be used to start answering such questions. This analysis incorporates biochemical and biophysical concepts to estimate some of the needed parameter values. We here leverage the theory against previously published single-live-cell experiments such as showcased in Figure 1. In this situation, human neutrophils act as uniquely capable biodetectors of minuscule amounts of anaphylatoxins produced by the host’s complement system at the surface of nearby bacterial, fungal, and model pathogens. The integration of theory and experiments allows us to pinpoint the primary role of complement-mediated chemotaxis within the overall hierarchy of immunotaxis.

## Results

We have organized the Section “Results” of this interdisciplinary study as follows. Section “Mathematical Prescription of the Spatiotemporal Distribution of Chemoattractant” presents and discusses the main theoretical results in broadly accessible terms. Section “Rapidly Forming Anaphylatoxic Cloud Signals to Immune Cells the Presence and Location of Nearby Bacterial, Fungal, and Model Pathogens” demonstrates the application of this theory by presenting an in-depth analysis of the spatiotemporal distribution of anaphylatoxins under near-physiological conditions. Section “Mathematical Derivations and Compact Visualization of the Model Predictions” provides the technical background of our mathematical derivations and should be of primary interest to theoreticians; this part is not required in order to understand or apply the results of the earlier sections.

### Mathematical Prescription of the Spatiotemporal Distribution of Chemoattractant

How should one picture the concentration profile of chemoattractant around a given source? This question concerns every researcher studying chemotaxis (23–26). Conflicting opinions about the steepness of this profile are easily reconciled: it simply depends—for example, on the geometry of the source, or the distance from it. Yet, accurate predictions of the shape of such profiles are non-trivial and remain scarce.

An advanced quantitative analysis will seek to characterize not only the static—or steady-state—distribution of chemoattractant but also the evolution of this distribution over time. On the other hand, descriptions that capture all details of dynamic *in vivo* profiles of chemoattractant are likely to be prohibitively complex. Even after introducing many simplifying assumptions, mathematical treatments of this type of problem tend to require software packages that solve partial differential equations numerically (19, 27, 28).

The purpose of the theoretical part of this study is to present instead an analytical solution that can be used with standard plotting software and conveys intuitive insight into the spatiotemporal distribution of chemoattractant. Such closed-form solutions have the additional advantage that their corroboration does not require detailed familiarity with the underlying derivations. Instead, one can verify the final expressions by making sure that they satisfy the original model equations. Above all, broad interdisciplinary trust in a mathematical theory, and understanding of its applicability, require maximum transparency about the mechanistic scenario that the model translates into math.

#### Mechanistic Scenario

Our mathematical model accounts for the production, diffusion, and deactivation of chemoattractant in the following one-dimensional scenario (Figure 2A). A spherical particle of radius *R* is the source of a time-varying, radially symmetric distribution of chemoattractant. Starting at time *t* = 0, the chemoattractant is continually produced at the surface of this particle at a constant rate. The production rate is given as the number *j*_0_ of molecules produced per unit source-surface area per unit time. More precisely, *j*_0_ is a constant outward source flux that only exists directly at the particle surface.

The chemoattractant is redistributed in the surrounding infinite space by diffusion. We denote by *D* the diffusion coefficient of chemoattractant molecules in the given medium. Everywhere in this space, the chemoattractant is also deactivated or removed by an irreversible reaction that has the kinetic off-rate constant *k*. This removal is vital for many immunogenic chemoattractants because it prevents shock or overstimulation of the host organism. Such overstimulation would otherwise endanger the host by promoting autoimmune diseases or inflammatory disorders.

We denote the sought time-dependent radial concentration profile by *c*(Δ*r,t*), where Δ*r* = *r* − *R* is the radial distance from the surface of the source (Figure 2A). The concentration *c* is measured in units of number of molecules per volume. According to the above scenario, we initially have *c*(Δ*r*, 0) = 0. The buildup of *c*(Δ*r, t*) at times *t* > 0 depends on four parameters: *R, j*_0_, *D*, and *k*. The values of these parameters are defined by the particular experimental or physiological situation at hand.

A closely related scenario allows us to also predict what will happen after a source of chemoattractant disappears suddenly. Generally, a source particle that quickly moves away will disturb the concentration profile of chemoattractant in a manner that is too complex to describe analytically. We here consider instead the somewhat idealized scenario where the source particle disappears in an instant, leaving behind a space that is momentarily free of chemoattractant. In this case, the subsequent evolution of the diminishing concentration profile is primarily determined by the parameter values of *D* and *k* (and to some extent, *R*).

#### Ready-to-Use Analytical Solutions

**Figure 3:**
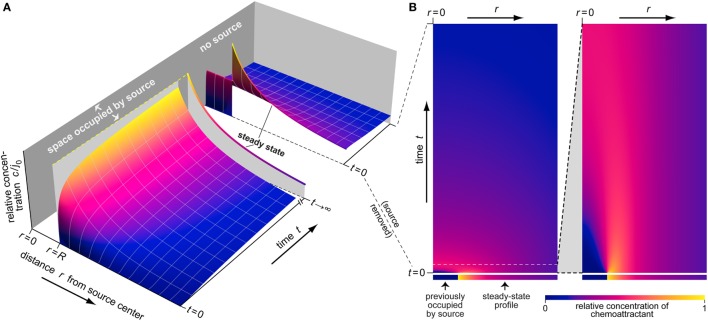
**Example of the buildup and dissipation of the spatiotemporal distribution of chemoattractant as predicted by Eqs 1–3**. **(A)** This 3D plot depicts three stages of the evolution of the relative concentration *c*/*j*_0_ (shown on the vertical axis). The first stage illustrates the buildup of the chemoattractant gradient around a source of radius *R*. The second part shows the steady-state concentration profile (at *t* →∞) as a single line. The third stage illustrates the dispersal of the chemoattractant after sudden removal of its source. **(B)** This density plot provides an alternative view of the dispersal of chemoattractant. Each horizontal line in this plot represents an instantaneous concentration profile at the respective time value. The ranges of the radial distance *r* and the time *t* in the first panel are the same as for the last stage of part **(A)**. The second panel presents a magnified view covering a small initial time interval.

For better readability of this paper, we have delegated the details of our mathematical derivations to the end of the Section “Results.” Here, we only list the final theoretical results, obtained for the mechanistic scenario described in the previous subsection. Figure 3 provides a representative overview of the type of information that this theory places at the fingertips of chemotaxis researchers.

The equation that predicts the buildup of the spatiotemporal concentration profile of chemoattractant in the presence of a source is found to be

(1) $$c\left(\Delta r,t\right)=\frac{j_{0}R^{2}}{\Delta r+R}\left\{\begin{array}{c} \frac{1}{2\left(D+R\sqrt{\mathrm{Dk}}\right)}\;\exp\;\left(-\frac{\Delta r}{\sqrt{D∕k\;}}\right)\;\text{erfc}\;\left(\frac{\Delta r}{2\sqrt{\mathrm{Dt}}}-\sqrt{\mathrm{kt}}\right)\\ +\frac{1}{2\left(D-R\sqrt{\mathrm{Dk}}\right)}\;\exp\;\left(\frac{\Delta r}{\sqrt{D∕k\;}}\right)\;\text{erfc}\;\left(\frac{\Delta r}{2\sqrt{\mathrm{Dt}}}+\sqrt{\mathrm{kt}}\right)\\ -\frac{1}{D-kR^{2}}\;\exp\;\left[\frac{\Delta r}{R}+\left(\frac{D}{R^{2}}-k\right)\;t\right]\;\text{erfc}\;\left(\frac{\Delta r}{2\sqrt{\mathrm{Dt}}}+\frac{\sqrt{\mathrm{Dt}}}{R}\right) \end{array}\right\}$$

Here, erfc denotes the complementary error function. The parameters that determine the time-varying concentration profile are the source radius *R*, the production rate (or source flux) *j*_0_, the diffusion coefficient *D*, and the removal rate constant *k*. For *t* = 0, Eq. 1 indeed reduces to the initial condition *c*(Δ*r*, 0) = 0.

In the steady state (where *t* → ∞ and ∂*c*/∂*t* = 0), Eq. 1 simplifies to

(2) $$c_{\infty }\left(\Delta r\right)={\left.c\left(\Delta r,t\right)\right|}_{t\to \infty }=\frac{j_{0}R^{2}}{\left(\Delta r+R\right)\left(D+R\sqrt{\mathrm{Dk}}\right)}\exp\left(-\frac{\Delta r}{\sqrt{D∕k\;}}\right)\;\text{for}\;k>0$$

As indicated in Eq. 2, this solution is only valid for non-zero values of *k*. If one disregards the removal reaction of chemoattractant, no steady state exists.

Equation 2 reveals that the steady-state concentration falls off rapidly as one moves away from the source. Its decline is determined by the product of two decaying functions, i.e., a 1/*r* dependence and an exponential decay. Such gradients are far from linear, which has important implications. First, conclusions drawn by previous studies that were based on the assumption of linear gradients must be taken with caution. Second, linear gradients created by microfluidic devices for chemotaxis studies are poor representations of physiological reality.

The behavior after the sudden removal of a source particle depends on the instantaneous concentration profile that existed at the moment of removal. We will here exclusively consider the case where, for *r* ≥ *R*, the new initial profile equals the steady-state profile given by Eq. 2. For *r* < *R*, the concentration of this initial profile equals 0, as explained in the previous subsection. For simplicity, we reset the clock to *t* = 0 at the moment of source removal. Then, the subsequent concentration profile is found to behave as

(3) $${\tilde{c}}_{0|\infty }\left(r,t\right)=\frac{1}{2}\frac{R^{2}j_{0}}{R\sqrt{\mathrm{Dk}}+D}\frac{1}{r}\times \left[\begin{array}{cc} & \exp\left(-\frac{r-R}{\sqrt{D∕k\;}}\right)\;\text{erfc}\;\left(-\frac{r-R}{2\sqrt{\mathrm{Dt}}}+\sqrt{\mathrm{kt}}\right)\\ & -\exp\left(\frac{r+R}{\sqrt{D∕k\;}}\right)\;\text{erfc}\;\left(\frac{r+R}{2\sqrt{\mathrm{Dt}}}+\sqrt{\mathrm{kt}}\right) \end{array}\right]$$

Here, we have introduced ${\tilde{c}}_{0|\infty }\left(r,t\right)$ to denote the time-varying concentration profile of chemoattractant after sudden removal of the source particle, provided that at the moment of removal (where *t* = 0) the concentration profile had reached the steady state. It is important to note that the concentration of Eq. 3 is expressed as a function of *r* rather than Δ*r*.

#### Parameter Effects

Figure 3 illustrates the dynamic behavior of an example concentration profile as predicted by Eqs 1–3. During the buildup phase, an early rapid rise of the concentration close to the source is followed by more gradual spreading of chemoattractant to larger distances. Eventually the distribution approaches a stationary profile that falls off rapidly at increasing radial distance (Figures 2A and 3A). After sudden removal of the source at a time where the steady-state concentration profile had been reached, the chemoattractant quickly dissipates, as shown in greater detail in Figure 3B.

The concentration *c*(Δ*r, t*) depends on four parameters: *j*_0_, *R, D*, and *k*. It is directly proportional to *j*_0_, the rate of production of chemoattractant per unit surface area of the source. In other words, changes in *j*_0_ will simply rescale the concentration profile but leave its overall shape unchanged. This also means that without knowledge of the source strength we cannot predict the absolute concentration and vice versa. If *j*_0_ is unknown, one can instead consider the relative concentration *c*/*j*_0_, which is the quantity shown in several figures of this paper.

The remaining three parameters affect *c*(Δ*r, t*) in a less tractable fashion. Because the source size *R* appears as another scaling term of the concentration, we generally expect larger sources to generate higher concentrations of chemoattractant. However, *R* also influences the actual shape of the concentration profile. To gain a better understanding of the effects of *R, D*, and *k*, it is more instructive to consider special cases such as the steady state or specific practical situations in which one or more parameter values are known. The applied section of this study presents a detailed discussion of the predictions of Eqs 1–3 for realistic estimates of the values of *D* and *k* in a physiologically important case.

Turning to the steady state and inspecting the signs of the partial derivatives of *c*_∞_(Eq. 2) with respect to *R, D*, and *k*, we find that an increase of the source size *R* will raise the steady-state concentration of chemoattractant everywhere. Conversely, an increase of the removal rate constant *k* will lower this concentration at any distance Δ*r*. The effect of the diffusion coefficient *D* is distance dependent. Higher diffusivity leads to lower concentrations near the source and to higher concentrations beyond a certain distance.

It is worth recalling that both the diffusion coefficient *D* and the kinetic rate constant *k* are inversely proportional to the viscosity of the surrounding fluid. The first relation follows from the Stokes-Einstein equation (Eq. 4), and the second is a result of Kramers’ reaction rate theory. Thus, as long as the environment behaves like a fluid, a change of environment will have little effect on the ratio *D*/*k*. Considering this ratio as constant, we find that the only remaining impact of the environment is an inverse proportionality of the form *c*_∞_ ∝1/*D*. Hence, a more viscous environment (where *D* is lower) will result in a higher steady-state concentration of chemoattractant at any distance Δ*r*. This rescaling effect does not alter the overall shape of the steady-state concentration profile.

#### How to Use This Mathematical Model?

The theory developed in this paper enables researchers to predict the concentrations of chemoattractants at any distance from their respective sources. If one is only interested in cases where the concentration has reached a stationary value, the steady-state equation (Eq. 2) suffices. Despite the formidable appearance of Eqs 1 and 3, their use to predict the instantaneous local concentration of chemoattractant is also straightforward.

The reliability of such predictions depends on two factors: the degree to which the model scenario matches a given experimental or physiological situation, and the accuracy of the used parameter values. The mechanistic scenario underlying our model reproduces the experimental situation of Figure 1 almost perfectly. It should also capture many other situations reasonably well. It is important to bear in mind though that issues like non-negligible convection, non-constant rates of the production or removal of chemoattractant, or a restricted diffusion space (e.g., when the source rests on the chamber bottom) are likely to introduce discrepancies between quantitative model predictions and observations.

Of the four needed parameters, the source size *R* is, in most cases, easy to estimate by microscopic inspection. The diffusion coefficient *D* of the chemoattractant might be known, although it is important to keep in mind that it depends on the medium through which the molecules diffuse. In cells or tissues, *D* can be measured using techniques like fluorescence recovery after photobleaching. If the chemoattractant is suspended in a fluid environment, its diffusion coefficient can be estimated using the Stokes-Einstein equation

(4) $$D=\frac{k_{\text{B}}T}{6\;\pi r_{p}\eta }$$

where *k*_B_ is the Boltzmann constant, *T* the absolute temperature, *r_p_* the radius of a chemoattractant molecule (presumed to be spherical), and η the dynamic viscosity of the fluid medium.

The values of the production rate per unit surface area, *j*_0_, and of the kinetic deactivation rate constant, *k*, are usually harder to come by. If enzymatic reactions govern the production or removal of chemoattractant, suitable approximations might allow one to recast the relevant enzyme kinetics into the simplified forms used by our model. For example, a constant source flux *j*_0_ requires that the density of enzymes decorating the source surface be constant and that changes of the substrate concentration can be neglected. The rate of enzymatic deactivation of the chemoattractant can be cast into the form of −*kc* (as required by our model) if the concentration of chemoattractant is small (see the next section for an example). Of course, estimates of *j*_0_ or *k* based on such approximations require that the kinetic constants of the respective reactions as well as the values of enzyme and substrate concentrations be known. In other situations, one has to decide on a case-by-case basis to what extent chosen parameter values represent reality.

The next section presents a detailed example of the above strategy, applied to the case of anaphylatoxins produced by the host’s complement system at the surface of pathogens.

### Rapidly Forming Anaphylatoxic Cloud Signals to Immune Cells the Presence and Location of Nearby Bacterial, Fungal, and Model Pathogens

The experiments compiled in Figure 1 and Video S1 in Supplementary Material provide topical examples of the relevance of the theory developed in this paper, placing it into the context of a vital defense mechanism of the human immune system against pathogenic invaders (1–4). In these single live-cell experiments, we use micropipette manipulation and/or optical tweezers to maneuver target particles into the proximity of non-adherent, initially quiescent human neutrophils (17). Microscopic inspection of the resulting neutrophil morphology provides a clear readout of the chemotactic activity of the cells (Figure 2A). By supplementing autologous donor serum, we naturally reproduce the *in vivo* balance between the production and deactivation of chemoattractant.

In all cases where neutrophils extended chemotactic protrusions, this response required serum. Thus, N-formylated peptides could not have played a significant role in the recognition of bacterial targets. Instead, this type of chemotaxis was shown to be predominantly mediated by complement, in particular the anaphylatoxin C5a (1, 2).

C5a is an 11-kDa peptide of 74 amino acids. It is produced by the C5 convertase, an enzyme assembled by the host’s complement system on recognized pathogen surfaces (29–31). This convertase cleaves C5a from its precursor protein C5 downstream of the merging point of all three complement pathways (20, 21, 32). Once released from the pathogen surface, anaphylatoxins like C5a are quickly metabolized and deactivated by serum-based carboxypeptidases (22).

#### Experimental Observations Warranting an In-depth Quantitative Analysis

Whenever neutrophils detected a target, their responses exhibited similar features, marking complement-mediated chemotaxis as a universal mechanism of pathogen recognition that partakes in the human immune response to both bacteria as well as fungi. Common traits observed in hundreds of experiments included a fairly quick neutrophil response on a timescale of minutes, and a short range of recognition that was generally limited to 1–2 cell diameters. A quantitative analysis of one-on-one encounters between human neutrophils and zymosan particles reported a typical time lag of ~60 s between the placement of the target in the proximity of a cell and the first unambiguous sign of pseudopod formation (3). The same study measured a typical chemotaxis-initiation distance of ~5 μm, similar to the average diameter (~4–5 μm) of the used target particles. We note that the standard deviations of the results of both of these measurements were very large. For example, the apparent time lag from target placement to a cell morphology change ranged from as low as 6 s to several minutes.

As long as a given neutrophil was kept within a certain recognition distance from the target surface, the vigor of the chemotactic response did not appear to diminish over the time course of our experiments. This perseverance supports our assumption that target-bound convertases produce fresh anaphylatoxins continuously at a constant rate. On the other hand, if the target was removed beyond the recognition distance, the cell started retracting the pseudopod in a matter of seconds. (A demonstration of this behavior is included in example 5 of Video S1 in Supplementary Material.) These observations are clear evidence of the existence of a steady-state distribution of chemoattractant, i.e., a cloud of anaphylatoxins that persistently surrounds a stationary target. Moreover, they indicate that a threshold concentration of anaphylatoxins is needed to trigger and sustain the neutrophil response.

If—after an initial positive cell response—the target was relocated to a different side of the neutrophil, the resulting cell behavior depended on how exactly the target was moved. In cases where we moved the target in a wide arc, the cell responded by starting to retract the former pseudopod within seconds and forming a new pseudopod toward the repositioned target (Video S1 in Supplementary Material). In contrast, if the target was kept within the recognition distance while it was slowly repositioned, the pseudopod appeared to gradually follow the target motion. These observations reinforce the existence of a steady state and manifest the swiftness of the formation and dispersion of the spatial distribution of chemoattractant.

It is worth taking a moment to accentuate the fresh and unconventional perspective presented here. With few exceptions, conventional chemotaxis assays generate chemoattractant gradients to study the behavior of the responding cells. We here reverse this strategy by applying our preestablished knowledge of the cellular response to map out important features of the anaphylatoxic cloud. In other words, we employ individual human immune cells as *biodetectors* of anaphylatoxins produced at the surface of pathogens. Currently, there seem to be no other techniques that can detect these chemicals near individual target particles with a similar sensitivity. Figures 1 and 2B illustrate how this approach has allowed us to discriminate whether or not 11 different pathogen surfaces are able to evade recognition by complement-mediated chemotaxis. But how exactly should one picture the anaphylatoxic cloud in cases where a pathogen is recognized? What is the spatial reach of this cloud? Just how quickly does it form or disperse? How does it compare to the spatiotemporal distributions of other chemoattractants? It seems difficult to address such questions experimentally. Fortunately, the single-cell approach featured in Figure 1 and Video S1 in Supplementary Material lends itself to a detailed quantitative analysis using the theory developed in this study.

In the following subsections, we leverage our theory against the results of our reductionist experimental approach. We note that in all pure chemotaxis experiments, we had suspended the targets in serum-containing buffer for at least 1 h prior to their placement near a neutrophil. Therefore, it is safe to assume that the complement machinery—including the C5 convertase (29–31)—was fully assembled on the target surface at the start of each chemotaxis experiment.

To bolster the relevance of our theoretical predictions, we first estimate the values of some of the needed parameters, i.e., the diffusion coefficient *D* of the chemoattractant in the used buffer, as well as the rate constant *k* of the proteolytic deactivation of the chemoattractant.

#### Diffusion Coefficient of C5a in the Experiment Buffer

The diffusion coefficient depends on the molecule size, the buffer viscosity, and the temperature (Eq. 4). We assume that the peptide C5a is the main constituent of the cloud of anaphylatoxins that forms around a target in the presence of serum. Approximating the peptide as a sphere, we estimate its radius *r_p_* from its molecular weight MW using the formula

(5) $$\frac{r_{p}}{\left[\text{nm}\right]}\approx 0.066{\left(\frac{\text{MW}}{\left[\text{g}∕\text{mol}\;\right]}\right)}^{1∕3\;}$$

[established in Ref. (33); the appropriate units are indicated in square brackets]. For MW = 11 kDa, we find the peptide radius to be *r_p_* ≈ 1.5 nm.

The buffer used in the experiments contained 10–20% autologous donor serum. To determine the viscosity of this buffer accurately, we have designed a viscometer that can be used with minuscule amounts of test solution. We here used fluid volumes of 75 μL, but the same method can be applied to volumes as small as ~1 μL.

**Figure 4:**
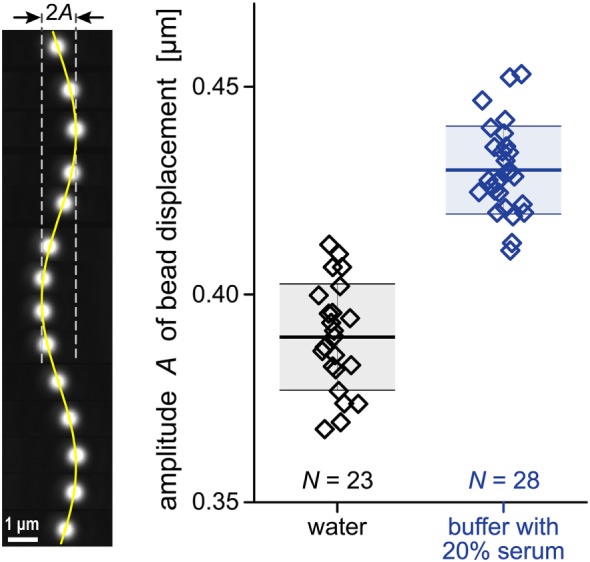
**Raw data for our viscosity measurement**. A periodic drag force was applied to cause sinusoidal displacements of polystyrene beads (~2.6 μm diameter) trapped by optical tweezers. Our analysis of such bead displacements is illustrated on the left. The vertical filmstrip comprises a sequence of 14 individual bead images taken at ~0.1 s intervals. The composite image includes a graph of a sine function representing the bead position as a function of time. The column-scatter plot on the right shows the amplitudes *A* of bead displacements measured in two different fluids. The numbers *N* of individual experiments are given in the plot. Thick horizontal lines mark the average amplitudes for each fluid. They are flanked by thinner lines showing the respective standard deviations. The numerical values of these quantities are given in Eq. 8.

The basic idea for this viscometer builds on a common approach to calibrate the spring constant *k_s_* of optical tweezers. It leverages the optical trapping force against an applied drag force generated by fluid flow past a laser-trapped microsphere. According to Stokes’ law, the drag force exerted by laminar flow on a spherical particle is proportional to the particle radius *R_b_*, the viscosity of the fluid η, and the relative flow velocity, i.e., the difference between the velocities of the surrounding fluid and the bead. In our measurements, flow past the laser-confined bead is generated by translating a piezoelectric microscope stage in a sinusoidal motion with amplitude *A*_0_ and frequency *f*_0_. The resulting drag force causes small sinusoidal bead displacements (Figure 4). The analysis of this linear system predicts that the amplitude *A*_buffer_ of the bead motion in a given buffer is

(6) $$A_{\text{buffer}}=\frac{A_{0}}{\sqrt{1+{\left(\frac{k_{s}}{12\pi^{2}f_{0}R_{b}\eta }\right)}^{2}}}\approx A_{0}\frac{12\pi^{2}f_{0}R_{b}\eta }{k_{s}}$$

For the settings used in our experiments, the term in parentheses is large compared to 1; therefore, we can approximate *A*_buffer_ as shown in the rightmost expression of Eq. 6.

We could use Eq. 6 to measure the viscosity η directly, but this would require that the values of all other quantities entering this equation be accurately known. Alternatively, we can determine the buffer viscosity relative to a standard solution like water. In this case, we measure both *A*_buffer_ and *A*_water_ under identical conditions. Then, Eq. 6 predicts that the ratio of *A*_buffer_/*A*_water_ is simply

(7) $$\frac{A_{\text{buffer}}}{A_{\text{water}}}=\frac{\eta_{\text{buffer}}}{\eta_{\text{water}}}\;\to \;\eta_{\text{buffer}}=\frac{A_{\text{buffer}}}{A_{\text{water}}}\eta_{\text{water}}$$

Previously developed algorithms (34) allow us to track the bead position with a resolution of a few nanometers. The polystyrene beads used for our viscosity measurements were fairly uniform but not identical in size, which caused some scatter in the amplitude data (Figure 4). We repeated the measurement for each of the two solutions with more than 20 beads. This gave (see also Figure 4)

(8) $$A_{\text{water}}=0.39\pm 0.01\left(\text{SD}\right)\;\mu \text{m}\;\text{and}\;A_{\text{buffer}}=0.43\pm 0.01\left(\text{SD}\right)\;\mu \text{m}$$

All measurements were performed at 20°C where water has a viscosity of η_water_ = 1 mPa⋅s. Thus, Eqs 7 and 8 yield the buffer viscosity η_buffer_ = 1.1 mPa⋅s.

With these values of the peptide radius and buffer viscosity, the Stokes-Einstein equation (Eq. 4) gives a diffusion coefficient of C5a of *D* ≈ 130 μm^2^ s^−1^.

#### Kinetic Off-Rate Constant for the Removal Reaction of C5a in the Experiment Buffer

According to quasi-steady-state Michaelis–Menten kinetics, the rate of enzymatic deactivation of C5a is given by

(9) $$-\frac{k_{\text{cat}}{\text{[}E\text{]}}_{0}c}{K_{M}+c}\;\overset{\text{if }c\;\ll \;K_{M}}{\to }\;\approx -\frac{k_{\text{cat}}{\text{[}E\text{]}}_{0}}{\underset{=k}{\underset{⎵}{\;K_{M}\;}}}c=-kc$$

where *c* is the concentration of the substrate C5a, [*E*]_0_ is the total concentration of the protease that deactivates C5a, and *k*_cat_ and *K*_M_ denote the turnover number and Michaelis constant of the enzyme, respectively. Assuming that the concentration of C5a always remains small compared to *K*_M_, we can linearize this expression as shown in Eq. 9. A first-order Taylor expansion simplifies the deactivation rate to the form −*kc*, as used in Eq. 10, which allows us to approximate the rate constant of the irreversible removal reaction of C5a by $k\approx k_{\text{cat}}{\left[E\right]}_{0}∕K_{\text{M}}\;$.

Several carboxypeptidases could be involved in the deactivation of C5a (22, 35–38), but it appears that the main serum-based “anaphylatoxin inactivator” (35) is carboxypeptidase N (22, 36). Its molecular weight is ~280 kDa, and its physiological concentration ~30 μg mL^−1^, or ~1.1 × 10^−7 ^M (22). Thus, in chemotaxis experiments that were conducted in buffer containing 20% autologous serum, the enzyme concentration was at least [*E*]_0_ ≈ 2.2 × 10^−8 ^M. The effective protease concentration could be higher because other carboxypeptidases might participate in the deactivation of C5a.

Reported values for the kinetic parameters *k*_cat_ and *K*_M_ of carboxypeptidase N depend on the substrate. For substrate peptides that—like C5a—have arginine at the C-terminus, typical *k*_cat_ values range from 4 to 139 s^−1^, and *K*_M_ values from 0.19 × 10^−4^ to 6.5 × 10^−4 ^M (39, 40). The measured ratios of *k*_cat_/*K*_M_ fall into the range from 6.7 × 10^3^ to 496.7 × 10^3 ^M^−1 ^s^−1^ (39, 40). To compensate for the fact that our estimate of [*E*]_0_ is a lower limit, we choose the high the value of *k*_cat_/*K*_M_ ≈ 5 × 10^5 ^M^−1 ^s^−1^ here. Together, this gives the rough estimate of the rate constant of the irreversible removal of C5a as *k* ≈ 1.1 × 10^−2 ^s^−1^.

#### Formation and Dispersion of the Anaphylatoxic Cloud

**Figure 5:**
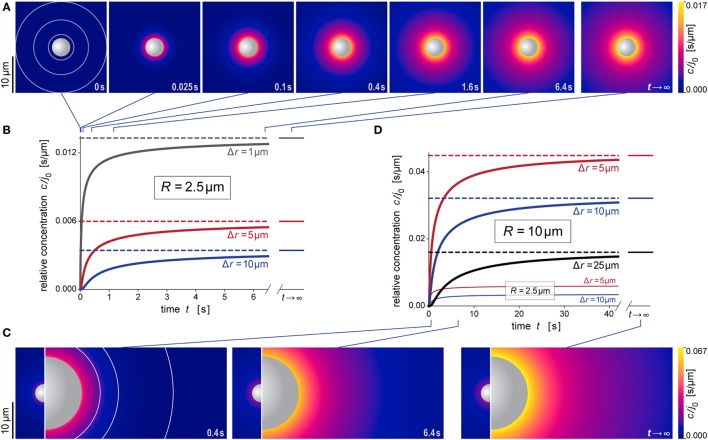
**Predicted behavior during formation of the anaphylatoxic cloud**. **(A)** Snapshots of our simulation of the evolution of the anaphylatoxic cloud around a spherical particle with radius *R* = 2.5 μm (see also Video S2 in Supplementary Material). The color gradient denotes the relative concentration *c*/*j*_0_. The concentric circles included in the first image indicate the distances from the source surface used for the plots in **(B)**. **(B)** Relative concentration *c*/*j*_0_ as a function of time at three selected distances Δ*r* for *R* = 2.5 μm. The time points corresponding to the density plots of **(A)** are indicated at the top. **(C)** Snapshots of our simulation of the evolution of the anaphylatoxic cloud around a spherical particle with radius *R* = 10 μm (see also Video S3 in Supplementary Material). The circle sections included in the first image indicate the distances from the source surface used for the plots in **(D)**. For direct comparison, each of the three panels includes the result obtained for *R* = 2.5 μm at the same time point. Here, the color gradients of all density plots use the same, common color scale. **(D)** Relative concentration *c*/*j*_0_ as a function of time at three selected distances Δ*r* for *R* = 10 μm. For direct comparison, two of the plots of **(B)** (obtained for *R* = 2.5 μm) are included as well. The time points corresponding to the density plots of **(C)** are indicated at the bottom. All concentrations were evaluated using the parameter values *D* = 130 μm^2^ s^−1^ and *k* = 1.1 × 10^−2^ s^−1^ typical for C5a.

**Figure 6:**
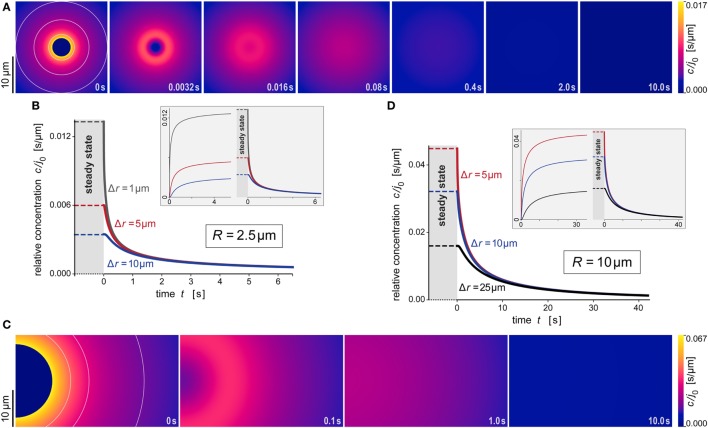
**Predicted behavior of the dispersing anaphylatoxic cloud after removal of the source**. **(A–D)** All panels are direct continuations of the respective panels of Figure 5, assuming that the source was suddenly removed at the newly reset time *t* = 0. The new initial profile was assumed to be given by Eq. 16, i.e., it was equal to the steady state for *r* ≥ *R* and zero for *r* < *R*. The insets in panels **(B,D)** include concentration curves of the earlier buildup of the anaphylatoxic cloud; these are the same as shown in Figures 5B,D. All concentrations were evaluated using the parameter values *D* = 130 μm^2^ s^−1^ and *k* = 1.1 × 10^−2 ^s^−1^ typical for C5a.

Assuming that the above estimates of *D* ≈ 130 μm^2^ s^−1^ and *k* ≈ 1.1 × 10^−2^ s^−1^ are typical for anaphylatoxins, and trusting that our model reliably captures the behavior of the anaphylatoxic cloud, we can use Eqs 1–3 to gain quantitative insight into this behavior that cannot be accessed by any other means. Because the sizes of bacterial, fungal, and surrogate targets vary, we will consider the source radius *R* as a control parameter, and examine in detail how it affects the spatiotemporal distribution of anaphylatoxins (Figures 5 and 6; Videos S2 and S3 in Supplementary Material). Unfortunately, the value of the fourth parameter, i.e., the source flux *j*_0_, is currently unknown. We hope to be able to estimate this value for various pathogen surfaces in the future, but as mentioned in Section “Parameter Effects,” our current predictions only consider the relative concentration *c*/*j*_0_.

Figures 5A,B and 6A,B illustrate the behavior of this relative concentration for *R* = 2.5 μm, the typical size of the smaller targets shown in Figure 1. The graphs reveal several important insights. First, the anaphylatoxic cloud forms rapidly around sources of this size (Figures 5A,B). It only takes a few seconds until the concentration reaches a plateau that is close to the steady state. Second, the spatial reach of the anaphylatoxic cloud remains small even in the steady state, i.e., its strength drops off quickly at a short distance from the source surface. Third, after sudden removal of the source, the anaphylatoxic cloud disperses very quickly (Figures 6A,B). In less than 1 s, the concentration profile becomes essentially flat, i.e., the gradient more or less disappears due to fast diffusion of the chemoattractant. Afterward, the lingering anaphylatoxins are gradually deactivated by carboxypeptidases.

Figures 5C,D and 6C,D highlight the strong dependence of *c*/*j*_0_ on the size of the source particle. These panels present predictions for a source with radius *R* = 10 μm. For direct comparison, Figures 5C,D also include results obtained with *R* = 2.5 μm (see also Figures 5A,B). The overall behavior during formation and dispersal of the anaphylatoxic cloud appears to be similar for the two target sizes, but it is important to note that the considered ranges of time, distance, and concentration are different between the two cases. The concentration around the larger source particle approaches the steady state more gradually (Figures 5C,D); however, even at a distance of Δ*r* = 25 μm from the surface of this larger source, the rising concentration surpasses in less than 5 s the steady-state concentration that is observed much closer (at Δ*r* ≈ 5 μm) to the smaller source particle (Figure 5D). After sudden removal of the source, it now takes a few seconds for the gradient to disappear (Figures 6C,D). When the concentration profile flattens out, the concentration of the remaining anaphylatoxins is considerable higher than for the smaller source; therefore, it is not surprising that the deactivation of the lingering chemoattractants now takes longer than for the smaller source.

**Figure 7:**
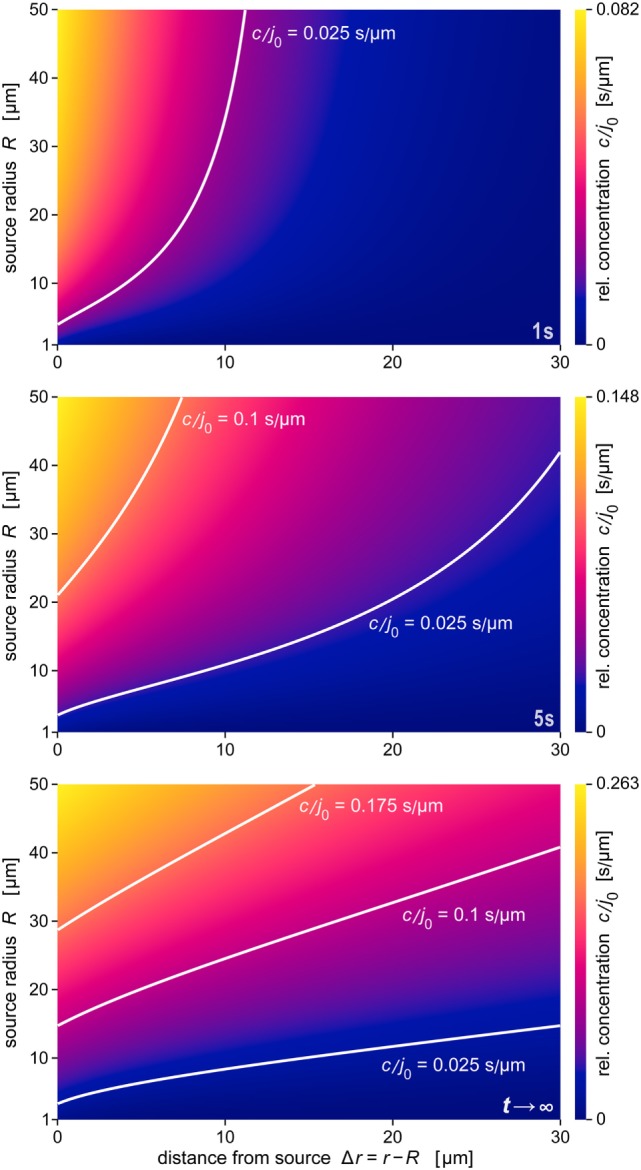
**Density maps of the relative concentration *c*/*j*_0_ as a function of Δ*r* and *R***. These maps were obtained at the three time points *t* = 1 s, *t* = 5 s, and *t* →∞ (steady state), respectively. Included are example contour lines of constant concentration (white curves). Note that the color scales of the three maps (shown at the right) are not the same. The data of all panels were evaluated using the parameter values *D* = 130 μm^2^ s^−1^ and *k* = 1.1 × 10^−2 ^s^−1^ typical for C5a.

**Figure 8:**
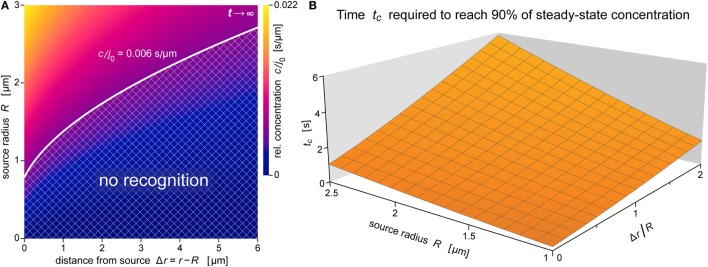
**Spatial reach and rate of formation of the anaphylatoxic cloud**. **(A)** Density map of the relative concentration *c*/*j*_0_ as a function of Δ*r* and *R* for *t* →∞ (steady state) for the ranges of source sizes and cell-target distances used in the experiments of Figure 1. Assuming that *c*/*j*_0_ = 0.006 s μm^−1^ is a typical threshold to trigger a neutrophil response, the corresponding white contour line separates a lower “no-recognition” region (cross-hatched) from the upper region where neutrophils are expected to respond to nearby pathogen particles by complement-mediated chemotaxis. **(B)** Characteristic approach time (defined by Eq. 18) to reach 90% of the steady-state concentration as a function of *R* and Δ*r*/*R*. All data were evaluated using the parameter values *D* = 130 μm^2^ s^−1^ and *k* = 1.1 × 10^−2 ^s^−1^ typical for C5a.

A comprehensive summary of the predicted behavior during the formation of the anaphylatoxic cloud is shown in Figure 7. The three panels map the relative concentration of chemoattractant as a function of the distance from the source and the source size at three time points. The color along horizontal lines in each density plot represents the radial concentration profile of chemoattractant for the respective value of *R*. Figure 7 also includes contour lines (white lines) of constant concentrations *c*/*j*_0_. Assuming that a threshold of chemoattractant concentration is needed to trigger a response by immune cells, the response distance of the cells as a function of the source size will be given by such a contour line (see also Figure 8A). The maps further emphasize the decisive role of the source size in determining the spatial reach of the chemoattractant cloud. In a sense, they show that being small can be viewed as a basic virulent factor of pathogenic microbes, because it furnishes them with protection against complement-mediated chemotactic recognition over moderate-to-large distances.

This notion is further illustrated by the concentration map of Figure 8A, which covers the typical ranges of source sizes and cell-target distances used in our experiments with smaller targets (Figure 1). A white contour line corresponding to *c*/*j*_0_ = 0.006 s μm^−1^ is superimposed as an example of a possible concentration threshold triggering a neutrophil response. We chose this value here because it roughly reproduces the chemotaxis-initiation distance of ~5 μm reported for zymosan particles (3). Assuming that this *c*/*j*_0_-value is typical for interactions between human neutrophils and various pathogen surfaces, the shown contour line subdivides the concentration map into two regions. Relative concentrations of anaphylatoxins that fall into the cross-hatched region below the white line are not expected to elicit a neutrophil response, whereas the region above this line comprises situations where our theory predicts a positive response by these cells. Remarkably, this graph predicts that source particles with diameters smaller than ~1.5 μm (where *R* ≤ ~0.75 μm)—which includes individual bacteria of *Salmonella* spp. and *E. coli*—are protected from complement-mediated, chemotactic recognition by human neutrophils, no matter how close they come to the surface of neutrophils. It is important to bear in mind though that this prediction relies strongly on the chosen threshold value of *c*/*j*_0_ and that it disregards any recognition that is likely to occur upon physical contact between cell and target.

The results of Figure 8A were obtained for the steady state. Figure 8B supplements this information with predictions of the time that it takes to approach the steady state. For source diameters between 2 and 5 μm, and cell-target distances ranging up to one source diameter, Figure 8B depicts the characteristic approach time *t_c_* defined by Eq. 18 (see the theory section below) for β − 1 = 0.9, i.e., for concentrations that equal 90% of the respective steady-state values. The 3D graph shows that throughout this region, the anaphylatoxic cloud reaches 90% of its maximum strength in less than 6 s, much faster than the typical response time of ~60 s reported for neutrophils encountering zymosan particles (3). Except for very small targets, the threshold concentration triggering a neutrophil response (cf. Figure 8A) is smaller than this 90% level, in which case the time to reach the cell activation threshold is even shorter than *t_c_*. Thus, Figure 8B confirms that the anaphylatoxic cloud forms rapidly in situations such as shown in Figure 1, and that the lag time observed before a visible neutrophil response is mainly due to intracellular processes.

#### Comparison of Theory and Experiments

We have not encountered any discrepancies between experimental observations and the theory presented here. The predicted small spatial reach of the anaphylatoxic cloud is consistent with the observed inability of neutrophils to recognize targets over distances larger than a certain threshold. This agreement corroborates our mechanistic explanation of the short recognition distance, i.e., the highly non-linear shape of the concentration profile caused by the balance of production, diffusion, and removal of chemoattractant.

Furthermore, the predicted rapid dispersal of the anaphylatoxic cloud after removal of the source is entirely consistent with the experimentally observed retraction of cellular pseudopods only seconds after a target particle is relocated. This agreement in dynamic behavior implies that signaling by the C5a receptor ceases very soon after the local concentration of its ligand drops below a certain threshold value.

On the other hand, the average delay between the formation of the anaphylatoxic cloud and the first visible sign of cell activation is so large that it can only be explained by an additional lag time required by the cell to start up its intracellular actin-remodeling machinery, in agreement with earlier reports (10, 41). Considering the natural cell-to-cell variability of live immune cells, this explanation is also consistent with the enormous spread of chemotaxis-initiation times reported in Ref. (3).

Our theory predicts that neutrophils need to get closer to smaller targets of a given type in order to recognize them. A recent experimental study has soundly validated this prediction, allowing us to quantify the sensitivity of human neutrophils to anaphylatoxins (4). The same effect predicts that targets smaller than a certain size limit will evade recognition via complement-mediated chemotaxis. This size threshold depends on the production rate per unit surface area, *j*_0_, which in turn depends on the chemical composition of the pathogen surface. Based on the rough estimate used in Figure 8A, we predict that human neutrophils are barely, if at all, able to recognize single bacteria of *Salmonella* Typhimurium and *E. coli* without physical contact. We have not systematically tested this prediction, but a careful review of our previous experiments (1) revealed that in a few cases where we had trapped *individual* bacteria with optical tweezers, they were indeed not recognized by nearby neutrophils without contact, no matter how small the cell-target distance was. Instead, positive chemotactic recognition always required a cluster of more than one bacterium.

Considering that our pure-chemotaxis experiments (e.g., Figures 1 and 2B and Video S1 in Supplementary Material) had not originally been designed for direct comparison with the theory developed here, the agreement between theory and experiments is remarkable. It conveys high confidence that the mechanistic scenario behind our model accurately captures the biophysical and biochemical processes governing the behavior of the anaphylatoxic cloud. This level of confidence gives rise to a picture of the anaphylatoxic cloud that, despite being inaccessible to direct experimental visualization, elevates our understanding from guesswork-based intuition to sound quantitative insight. As long as the source of anaphylatoxins moves sufficiently slowly, one may picture the 3D distribution of the chemoattractant as a relatively thin locator cloud that persistently surrounds the target.

### Mathematical Derivations and Compact Visualization of the Model Predictions

This theoretical section explains how we translate the known biophysical and biochemical mechanisms of chemotactic-gradient formation into math. It further provides the details of our solution of the resulting equations and investigates in general terms how parameter variations affect the predictions of this mathematical model. Although these technical details embody the main intellectual effort behind the current analysis, their in-depth understanding is not required to appreciate and apply the results of the previous subsections. Readers not interested in this theoretical background may safely skip the current section. We provide these details here to enable theoreticians to reproduce our main findings and, if needed, to adopt the math to other mechanistic scenarios than considered in this paper.

#### Mechanism-to-Math Translation and Solution

We denote the time-varying concentration profile of chemoattractant by $\tilde{c}\left(r,t\right)$. The tilde is used to emphasize that this concentration is a function of the radial distance *r* from the source center (Figure 2A). In contrast, when we express the concentration in terms of the distance Δ*r* = *r* − *R* from the source surface, we omit the tilde, i.e., $c\left(\Delta r,t\right)=\tilde{c}\left(r,t\right)=\tilde{c}\left(\Delta r+R,t\right)$.

The mechanistic scenario described in Section “Mechanistic Scenario” translates into the following mathematical form:

(10) $$\begin{array}{ll} \begin{array}{ccccc} & \frac{\partial \tilde{c}}{\partial t} & \;=D\left(\frac{2}{r}\frac{\partial \tilde{c}}{\partial r}+\frac{\partial^{2}\tilde{c}}{\partial r^{2}}\right)-k\tilde{c}\\ \text{ Initial condition} & \tilde{c}\left(r,0\right)\; & =0 & \text{ for}\;\; & R\leq r<\infty \\ \text{Boundary conditions} & {\left.\tilde{c}\left(r,t\right)\right|}_{r\to \infty }\; & =0 & \text{ for}\;\; & 0<t<\infty \\ & \text{and}{\left.\frac{\partial \tilde{c}\left(r,t\right)}{\partial r}\right|}_{r=R}\; & =-\frac{j_{0}}{D} & \text{ for}\;\; & 0<t<\infty \end{array} & \end{array}$$

Equation 10 describes a diffusion–reaction problem of radial symmetry that is somewhat complicated by the boundary condition for the concentration gradient at the source surface where *r* = *R*. This Neumann-type boundary condition is Fick’s first law; it incorporates the delta source flux while prohibiting net flux of chemoattractant into the sphere.

We first find the steady state of this problem by setting $\partial \tilde{c}∕\partial t\;=0$. Using the transformation $u\left(r,t\right)=r\tilde{c}\left(r,t\right)$, the resulting differential equation is readily solved to give Eq. 2.

To find the time-dependent solution, we transform Eq. 10 into an equation with constant coefficients, and place the source surface at the origin of the coordinate system of the resulting plane problem. Defining

(11) $$w\left(\Delta r,t\right)=u\left(\Delta r+R,t\right)=u\left(r,t\right)=r\;\tilde{c}\left(r,t\right)$$

Equation 10 becomes

(12) $$\begin{array}{ll} \begin{array}{rl} \frac{\partial w}{\partial t} & =D\frac{\partial^{2}w}{\partial \Delta r^{2}}-\mathrm{kw}\\ \text{Initial condition}\;w\;\left(\Delta r,0\right) & =0\;\text{ for}\;0\leq \Delta r<\infty \\ \text{Boundary conditions}\;{\left.w\;\left(\Delta r,t\right)\right|}_{\Delta r\to \infty } & =0\;\text{ for}\;0<t<\infty \\ \text{and}\;{\left.\left[\frac{\partial w\left(\Delta r,t\right)}{\partial \Delta r}-\frac{1}{R}w\left(\Delta r,t\right)\right]\right|}_{\Delta r=0} & =-\frac{j_{0}R}{D}\;\;\text{ for}\;\;0<t<\infty \end{array} & \end{array}$$

This transformation has converted the Neumann boundary condition of Eq. 10 into a Robin-type boundary condition. (Alternatively, we could have used Danckwert’s transformation method (42) that leaves the form of the boundary condition unaltered. However, this would result in the same overall mathematical effort because the back-transformation would require additional algebraic manipulation.)

The general solution of Eq. 12 in terms of the Green’s function is listed in Ref. (43). Adopting this solution to the current problem and evaluating all required integrals, we eventually arrive at the main theoretical result of this study given in Eq. 1. This solution indeed satisfies Eq. 10, and for t→∞ it reduces to the steady-state solution Eq. 2.

As explained in Section “Ready-to-Use Analytical Solutions,” the behavior after sudden removal of the source particle depends on the instantaneous concentration profile that existed at the moment of removal. Denoting this new initial profile by ${\tilde{c}}_{0}\left(r\right)$, the subsequent concentration of chemoattractant is the solution of the problem

(13) $$\begin{array}{ll} \begin{array}{cccccc} \; & \; & \frac{\partial \tilde{c}}{\partial t}\; & =D\left(\frac{2}{r}\frac{\partial \tilde{c}}{\partial r}+\frac{\partial^{2}\tilde{c}}{\partial r^{2}}\right)-k\tilde{c}\\ \;\\ \; & \text{Initial condition}\; & \tilde{c}\left(r,0\right)\; & ={\tilde{c}}_{0}\left(r\right) & \text{ for}\;\;\;\; & 0\leq r<\infty \\ \;\\ \; & \text{Boundary condition}\; & {\left.\frac{\partial \tilde{c}\left(r,t\right)}{\partial r}\right|}_{r=0}\; & =0 & \text{ for}\;\;\;\; & 0<t<\infty \end{array} & \end{array}$$

Note that the clock is reset to *t* = 0 at the moment of source removal. Changing variables from $\tilde{c}\left(r,t\right)$ to $u\left(r,t\right)$ via the transformation Eq. 11, this becomes

(14) $$\begin{array}{ll} \begin{array}{rl} \frac{\partial u}{\partial t} & =D\frac{\partial^{2}u}{\partial r^{2}}-\mathrm{ku}\\ \text{Initial condition}\;u\left(r,0\right) & =r{\tilde{c}}_{0}\left(r\right)\;\text{for}\;0\leq r<\infty \\ \text{Boundary condition}\;{\left.\left[\frac{\partial u\left(r,t\right)}{\partial r}-\frac{1}{r}u\left(r,t\right)\right]\right|}_{r=0} & =0\;\text{for}\;0<t<\infty \end{array} & \end{array}$$

After some algebra, the general solution of this problem [given in Ref. (43)] can be simplified to

(15) $${\tilde{c}}_{0|t}\left(r,t\right)=\frac{\exp\;\left(-\mathrm{kt}\right)}{2r\sqrt{\pi \mathrm{Dt}}}\int_{0}^{\infty }\rho \;{\tilde{c}}_{0}\left(\rho \right)\times \left\{\exp\;\left[-\frac{{\left(r-\rho \right)}^{2}}{4\mathrm{Dt}}\right]-\exp\;\left[-\frac{{\left(r+\rho \right)}^{2}}{4\mathrm{Dt}}\right]\right\}\text{d}\rho$$

In this study, we exclusively consider the case where ${\tilde{c}}_{0}\left(r\right)$ is determined by the steady-state profile given in Eq. 2, i.e.,

(16) $${\tilde{c}}_{0}\left(r\right)=\left\{\begin{array}{ll} c_{\infty }\left(r-R\right)\; & \text{if}\;r\geq R\\ 0\; & \text{if}\;0\leq r<R \end{array}\right.$$

In this case, the integral in Eq. 15 can be evaluated to give Eq. 3.

#### Spatial Extent of the Steady-State Cloud of Chemoattractant Surrounding the Source

Although the concentration profile extends to infinity at all times *t* > 0, its steady-state shape resembles a cloud of chemoattractant that thins out quickly as one moves away from the source (Figures 2A and 3A). The drop in concentration is governed by two contributions: an exponential decay, and a multiplicative 1/*r* term (Eq. 2). We define as the characteristic width of this cloud the value Δ*r_c_* at which the concentration *c*_∞_(Δ*r*) has dropped to a fraction of α (0 ≤ α ≤ 1) times the concentration at the source surface, *c*_∞_(0). Thus, Δ*r_c_* is given by

(17) $$\frac{c_{\infty }\left(\Delta r_{c}\right)}{c_{\infty }\left(0\right)}=\alpha \;\to \;\Delta r_{c}=-R+\lambda W_{0}\;\left(\frac{1}{\alpha }\frac{R}{\lambda }\;\exp\;\left[\frac{R}{\lambda }\right]\;\right),\lambda \equiv \sqrt{D∕k\;}$$

where *W*_0_(*x*) denotes the principal branch of the Lambert *W* function, and we have introduced the abbreviation $\lambda =\sqrt{D∕k}$. The parameter λ is the decay length of the exponential function appearing in Eq. 2. It is proportional to the typical distance traversed by a diffusing molecule of chemoattractant during its average lifetime. As mentioned in Section “Parameter Effects,” λ is largely independent of the fluid environment in which the source is immersed.

It is important to bear in mind that the characteristic width Δ*r_c_* refers to a normalized concentration profile whose value at the source surface always equals 1. The purpose of Eq. 17 is to define a single quantity to assess the overall shape of the chemoattractant cloud. Small Δ*r_c_* values characterize steeply decaying concentration profiles, whereas larger values of Δ*r_c_* correspond to more gradually declining profiles.

**Figure 9:**
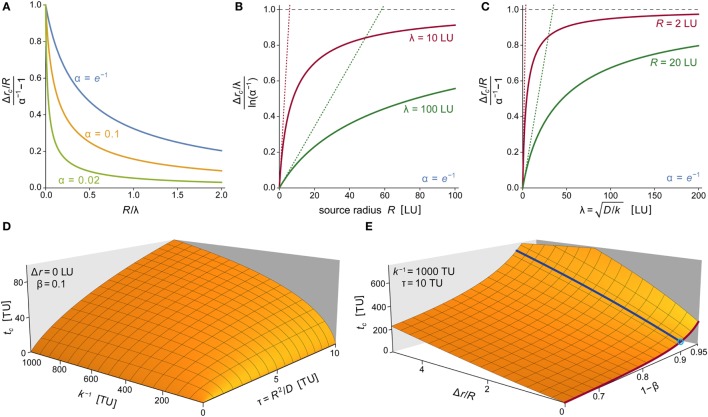
**Parameter dependence of chemoattractant cloud**. **(A)** Plots of the normalized characteristic width of the chemoattractant cloud as a function of *R*/λ for three values of α. The cloud thickness Δ*r_c_* is given by Eq. 17, which also defines λ = (*D*/*k*)^1/2^. Here, Δ*r_c_* has been normalized with respect to its limiting value at *R*/λ →0, which equals *R*(α^−1 ^−^ ^1). **(B)** Plots of the normalized characteristic width of the chemoattractant cloud as a function of *R* for two values of λ. Here, Δ*r_c_* has been normalized with respect to its limiting value at *R* →∞, which equals ρ_∞_ = λln(α^−1^). Dotted straight lines represent linearized versions of the respective solid curves (obtained by a Taylor expansion at *R* = 0; with slopes of (α^−1^ − 1)/ρ_∞_). **(C)** Plots of the normalized characteristic width of the chemoattractant cloud as a function of λ for two values of *R*. Here, Δ*r_c_* has been normalized with respect to its limiting value at λ →∞, which equals ρ_∞_ = *R*(α^−1^ − 1). Dotted straight lines represent linearized versions of the respective solid curves (obtained by a Taylor expansion at λ = 0; with slopes of ln(α^−1^)/ρ_∞_). **(D)** 3D plot of the characteristic time *t_c_* of the approach to the steady state of the chemoattractant cloud as a function of the two timescales *k*^−1^ and τ = *R*^2^/*D*. The approach time *t_c_* shown here is the numerical solution of Eq. 18 for β = 0.1 and Δ*r* = 0. **(E)** 3D plot of the characteristic approach time *t_c_* as a function of Δ*r*/*R* and 1 − β for *k*^−1^ = 1,000 TU and τ = 10 TU. Superimposed are two solid lines obtained for Δ*r*/*R* = 0 (dark red line) and 1 − β = 0.9 (dark blue line). The crossing of these two lines (marked by a small circle) corresponds to the parameter values used in panel **(D)**. In several panels, we have used “LU” and “TU” to denote generic length and time units, respectively. These plots are valid for any real units chosen to replace LU and TU as long as all instances of the generic units are replaced by the same respective real units.

Figure 9A presents a compact visualization of Eq. 17 in dimensionless form. Although the choice of α affects the relationship between Δ*r_c_* and the two length scales *R* and λ, it does not change the overall trend of this relationship. Figures 9B,C depict examples of the monotonously increasing dependences of Δ*r_c_* on *R* and λ, respectively. The graphs confirm that larger source sizes, higher diffusion coefficients, or longer molecular lifetimes (i.e., smaller values of *k*) act to increase the spatial reach of the chemoattractant cloud. Moreover, for small values of *R* or λ, the characteristic width Δ*r_c_* is highly sensitive to changes of these parameters, whereas at larger values of *R* or λ, Δ*r_c_* approaches a plateau and becomes more robust with respect to parameter variations.

#### Dynamics of the Formation of the Chemoattractant Cloud

To assess the practical relevance of steady-state predictions, we need to know how quickly the steady state is approached in comparison with typical experiment times. Any estimate of the rate of approach to the steady state depends on the distance Δ*r* at which it is evaluated. Keeping this dependence in mind, we define as a characteristic measure of the time required to reach the steady state the time *t_c_* at which the concentration of chemoattractant at a particular distance Δ*r* has risen to a fraction of (1 − β) times its steady-state value. Thus for given Δ*r* and 0 ≤ β ≤ 1, the characteristic approach time *t_c_* is the solution of the equation

(18) $$\frac{c\left(\Delta r,t_{c}\right)}{c_{\infty }\left(\Delta r\right)}=1-\beta$$

For example, if we choose to evaluate *t_c_* at the source surface (Δ*r* = 0), *t_c_* will be given by

(19) $$\beta =\frac{1}{1-\sqrt{k\tau }}\left\{\exp\;\left[\left(\frac{1}{\tau }-k\right)t_{c}\right]\;\text{erfc}\;\left(\sqrt{\frac{t_{c}}{\tau }}\right)-\sqrt{k\tau }\;\text{erfc}\;\left(\sqrt{kt_{c}}\right)\right\},\;\tau \equiv R^{2}∕D\;$$

The timescale τ = *R*^2^/*D* introduced in Eq. 19 is proportional to the typical time that it takes a molecule of chemoattractant to traverse an average distance of *R* by diffusion in the absence of a removal process.

Equation 19 reveals that the characteristic time *t_c_* required to reach the steady state at Δ*r* = 0 depends on two parameters: the diffusion timescale τ and the typical lifetime *k*^−1^ of a molecule of chemoattractant. For Δ*r* > 0, Eq. 18 can be rewritten in terms of the same two timescales and the normalized distance Δ*r*/*R*. The numerical solution of Eq. 18 allows us to examine graphically how *t_c_* depends on τ and *k*^−1^ (Figure 9D) as well as on our choices of Δ*r*/*R* and β (Figure 9E).

## Discussion

The ability of chemotaxing immune cells to prioritize their responses to different stimuli is critical to an efficient immune response in health and disease (2, 11, 14, 15). The primary cause of a cellular switch between different chemotactic responses is a change in the relative strengths of chemotactic stimuli encountered at the cell surface. An intriguing intracellular signaling hierarchy can then reinforce the cellular decision, solidifying the commitment of the cell to one type of chemotactic response over another. The latter mechanism has been thoroughly examined using the under-agarose assay (6). Traditional experimental methods are poorly suited, however, to assess the local strengths of chemotactic stimuli. Instead, theoretical approaches are needed to characterize the original cause of the switch between chemotactic responses. The central task of such simulations is the reliable prediction of the spatiotemporal distributions of chemoattractants around their sources. By addressing this task in a broadly accessible manner, the present study adds a new, quantitative dimension to the investigation of immunotaxis.

In-depth quantitative analyses of the time-varying concentration profiles of chemoattractants are non-trivial and remain scarce. Previous studies have tackled this challenge using numerical methods (27, 28). Such methods are often necessary, but their use requires special training, and their results provide limited intuition about the general behavior of the studied scenarios. Overcoming these limitations, we have instead developed a closed-form mathematical prescription of the evolution of chemoattractant gradients. The manner in which we model the production and deactivation of chemoattractant closely aligns with physiological reality as well as with our single-cell, pure-chemotaxis experiments. Of particular importance is the inclusion of the removal reaction of chemoattractant. It represents a simple mathematical equivalent of vital physiological mechanisms that prevent overstimulation of the host organism by chemoattractant.

It is important to bear in mind that the convenience of an analytical solution comes at the cost of simplifying assumptions. The validity of these assumptions must be checked on a case-by-case basis. The diffusion–reaction scenario underlying our analytical solution disregards convection, and it assumes that the chemoattractant source flux (or production rate), as well as the removal rate constant, remain unchanged on the considered timescale. These assumptions appear to be valid in the case of our pure-chemotaxis experiments, and they should be reasonable for many biological situations.

It is worth commenting on the distinction between the concentration threshold that triggers the chemotactic activation of a responding cell and the gradient that defines the cell’s direction of motion. Naturally, the former is a prerequisite of the cell’s decision how to steer a forming pseudopod. The present analysis only deals with the former issue, considering the critical concentration local to the front of the cell (where the distance to the source is shortest). However, our theoretical predictions should be just as useful for estimates of the time-dependent chemoattractant gradient experienced by a cell, i.e., the varying concentration of chemoattractant along the cell surface (44). Such insight is critical for quantitative studies of gradient sensing and cell polarization. Similarly, knowledge of the local concentrations of the ligands of G-protein-coupled receptors is an important prerequisite of quantitative studies of GPCR function.

These potential applications illustrate the general usefulness of our theory. In this study, we have applied it to analyze the spatiotemporal distribution of anaphylatoxins around pathogenic targets. Anaphylatoxins and the popular *in vitro* stimulant N-formylmethionyl-leucyl-phenylalanine (fMLP) are often indiscriminately categorized as end-target-derived chemoattractants. Our single-cell experiments have refined this notion by identifying anaphylatoxins as the main mediator of neutrophil chemotaxis to live bacteria (1). Thus, taking into account the well-established role of anaphylatoxins in the chemotactic recognition of fungi, we conclude that complement-mediated chemotaxis is, in fact, the predominant recognition mechanisms by which immune cells detect both fungal as well as live bacterial pathogens from a distance. This important role of anaphylatoxins calls for a deeper mechanistic understanding of their behavior, lending weight to our in-depth analysis of the anaphylatoxic cloud.

This analysis benefits greatly from the integration of theory and closely related experiments. Our integrative approach has not only provided mechanistic explanations for our experimental observations, it has also boosted confidence in the theoretical predictions. For example, there can be little doubt that carboxypeptidases play an important role in shaping highly non-linear gradients of anaphylatoxins like C5a *in vivo*—an effect that is often neglected by *in vitro* chemotaxis assays with C5a. Overall, our analysis paints a clear picture of the spatiotemporal distribution of anaphylatoxins as a rapidly forming, thin locator cloud that persistently surrounds stationary and slowly moving target particles. An interesting implication of this analysis is our prediction that simply being small is an effective protective means by which very small pathogenic targets (with sizes of up to ~1 μm) can avoid complement-mediated recognition from a distance.

Our results also rule out the possibility that complement-mediated chemotaxis could be involved in long-range recruitment of immune cells to targets of small-to-moderate sizes. Hence, other chemoattractants like intermediate chemokines have to be responsible for neutrophil recruitment over larger distances. To be effective over such distances without overstimulating the host, gradients of intermediate chemokines have to be shallower than the rapidly declining profile of anaphylatoxins. Once a newly recruited cell comes sufficiently close to a target that has been recognized by complement and is surrounded by a cloud of C5a, the cell is predicted to be exposed to a dramatic and fairly sharp increase of C5a levels. At some point, the highly non-linear gradient of anaphylatoxins is likely to cause the cell to abandon intermediate chemokines—a decision that is further reinforced by inhibition of the intracellular signals mediating the former, intermediate type of chemotaxis (6). These considerations show how our integrative approach allows us to pinpoint the role of complement-mediated chemotaxis within the paradigm of immunotaxis, i.e., as a universal, but short-range, homing mechanism by which chemotaxing immune cells can implement a last-minute course correction toward pathogenic microbes.

## Materials and Methods

The materials and methods used in our earlier experiments with bacterial, fungal, and model pathogens have been described in previous work (1–4).

To measure the buffer viscosity needed in the present analysis, we used spherical polystyrene particles with an average diameter of 2.6 μm (Spherotech, Inc., Lake Forest, IL, USA). These particles were diluted and used without further treatment. Viscosity measurements were performed using our previously described optical tweezers instrument (34).

## Author Contributions

VH developed the theory, carried out the simulations, designed the experiments, and prepared the manuscript. WS and EF performed experiments and analyzed the data.

## Conflict of Interest Statement

The authors declare that the research was conducted in the absence of any commercial or financial relationships that could be construed as a potential conflict of interest.

## Supplementary Material

Video S1 **Human neutrophils detect and chase bacterial and fungal pathogens by complement-mediated chemotaxis**.

Video S2 **Formation, steady state, and dispersal of the anaphylatoxic cloud**.

Video S3 **Dependence of the spatiotemporal distribution of chemoattractant on the size of the source**.
