# Supplementary material for: Analytical Prediction of the Spatiotemporal Distribution of Chemoattractants around Their Source: Theory and Application to Complement-Mediated Chemotaxis
Source: Front Immunol. 2017 May 26;8:578. doi: 10.3389/fimmu.2017.00578 (PMC5445147; doi:10.3389/fimmu.2017.00578)
Supplement: Supplementary file 4 [file Data_Sheet_1.PDF]

Supplementary Material

Analytical Prediction of the Spatiotemporal Distribution of Chemoattractants around their Source: Theory and Application to Complement-Mediated Chemotaxis

Volkmar Heinrich*, **Wooten D. Simpson III, Emmet A. Francis**

*** Correspondence:** Volkmar Heinrich: vheinrich@ucdavis.edu

1 Supplementary Video Legends with Thumbnails

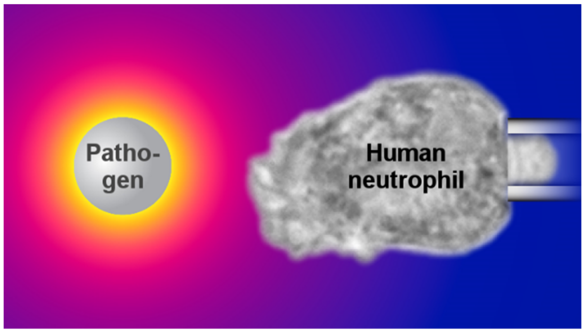

Supplementary Video 1. Human neutrophils detect and chase bacterial and fungal pathogens by complement-mediated chemotaxis. This video showcases six examples of pure-chemotaxis experiments testing for the short-distance recognition of various pathogens by human neutrophils. The video demonstrates the discriminative power of our single-cell assay by contrasting the vigorous neutrophil responses in the first five examples with the lack of chemotactic recognition of *C. neoformans* in the last example. A compilation of the results of chemotaxis tests with 11 different targets is included at the end. This type of experiment offers superb control over one-on-one encounters between immune cells and pathogenic targets. Positive responses are triple-checked by repositioning the target to different sides of the pipette-held cell using optical tweezers or a second micropipette. The clear evidence provided by the direct visualization of pure (i.e., unbiased by cell-substrate adhesion) chemotaxis obviates the need for the accumulation of circumstantial evidence. Integrated with innovative theory, this interdisciplinary approach allows us to address key mechanisms of vital cellular behavior that are inaccessible to traditional biological methods.

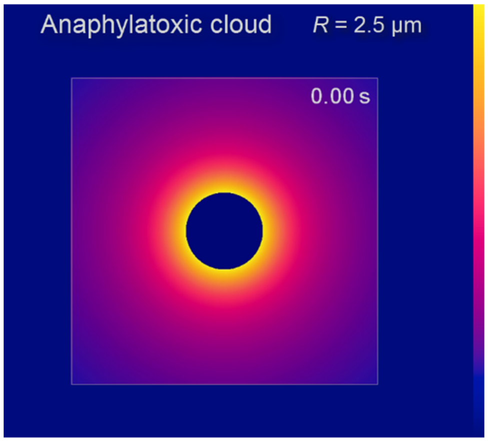

Supplementary Video 2. Formation, steady state, and dispersal of the anaphylatoxic cloud. The video shows a simulation of the time-dependent concentration profile of anaphylatoxins around a spherical source with radius $R = 2.5\,\mu\text{m}$. The relative concentration (depicted using the color scale included at the right) was calculated using a diffusion coefficient of $D = 130\,\mu\text{m}^2/\text{s}$ and a removal-rate constant of $k = 1.1\times10^{-2}\,\text{s}^{-1}$ typical for C5a in the presence of 20% serum. Under these conditions, the anaphylatoxic cloud rapidly approaches the steady state. After removal of the source, most of the chemoattractant disperses even faster. The spatial reach of the anaphylatoxic cloud remains small

even in the steady state ($t \to \infty$).

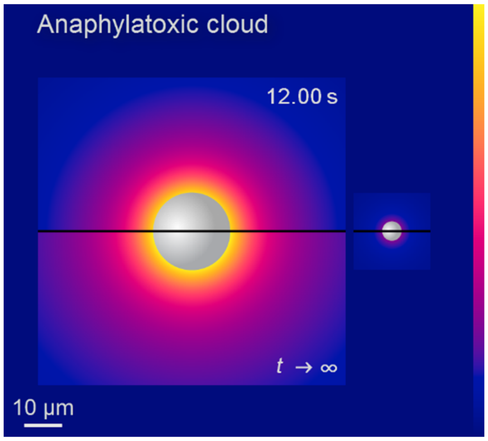

Supplementary Video 3. Dependence of the spatiotemporal distribution of chemoattractant on the size of the source. The video first shows a simulation of the formation, steady state, and dispersal of the anaphylatoxic cloud around a spherical source with radius $R = 10\,\mu\text{m}$ in real time. The second part presents a side-by-side comparison of the buildup of the concentration profiles of anaphylatoxins around spherical sources of two different sizes. Apart from the size difference, both simulations used identical conditions, in particular, a diffusion coefficient of $D = 130\,\mu\text{m}^2/\text{s}$ and a removal-rate constant of $k = 1.1 \times 10^{-2}\,\text{s}^{-1}$ typical for C5a in the presence of 20% serum. The relative concentration of chemoattractant is depicted using the same common color scale for both simulations. At the 12-s time point, the simulations are halted, and the steady state concentration profiles ($t \to \infty$) are shown in the respective lower parts of vertically split density plots.
